# Assessing the Potential of Brewer’s Spent Grain to Enhance Cookie Physicochemical and Nutritional Profiles

**DOI:** 10.3390/foods14010095

**Published:** 2025-01-02

**Authors:** Marisa Nicolai, Maria Lídia Palma, Ricardo Reis, Rúben Amaro, Jaime Fernandes, Elsa M. Gonçalves, Mafalda Silva, Manuela Lageiro, Adília Charmier, Elisabete Maurício, Patrícia Branco, Carla Palma, Joaquim Silva, Maria Cristiana Nunes, Pedro C. B. Fernandes, Paula Pereira

**Affiliations:** 1 CBIOS—Research Center for Biosciences & Health Technologies, Universidade Lusófona, Campo Grande 376, 1749-024 Lisboa, Portugal; lidia.palma@ulusofona.pt (M.L.P.); elisabete.mauricio@ulusofona.pt (E.M.); paula.pereira@ulusofona.pt (P.P.); 2 EPCV, School of Psycology and Life Science, Department of Live Sciences, Universidade Lusófona, Campo Grande 376, 1749-024 Lisboa, Portugal; ricardoreis19102003@gmail.com (R.R.);; 3 INIAV—Instituto Nacional de Investigação Agrária e Veterinária, Unidade de Tecnologia e Inovação, 2780-157 Oeiras, Portugal; jaime.fernandes@iniav.pt (J.F.); elsa.goncalves@iniav.pt (E.M.G.); manuela.lageiro@iniav.pt (M.L.); 4 GeoBioTec—Geobiociências, Geoengenharias e Geotecnologias, Faculdade de Ciências e Tecnologia, Universidade NOVA de Lisboa, 2829-516 Caparica, Portugal; 5 BioRG—Bioengineering and Sustainability Research Group, Faculty of Engineering, Universidade Lusófona, Campo Grande 376, 1749-024 Lisboa, Portugal; adilia.charmier@ulusofona.pt (A.C.); patricia.branco@ulusofona.pt (P.B.); joaquim.silva@ulusofona.pt (J.S.); pedro.fernandes@ulusofona.pt (P.C.B.F.); 6 LEAF—Linking Landscape, Environment, Agriculture and Food Research Center, Associate Laboratory TERRA, Instituto Superior de Agronomia, Universidade de Lisboa, Tapada da Ajuda, 1349-017 Lisboa, Portugal; cristiananunes@isa.ulisboa.pt; 7 Instituto Hidrográfico, Rua das Trinas 49, 1249-093 Lisboa, Portugal; carla.palma@hidrografico.pt; 8 iBB—Institute for Bioengineering and Biosciences, Instituto Superior Técnico (IST), Universidade de Lisboa, Av. Rovisco Pais, 1049-001 Lisbon, Portugal; 9 Associate Laboratory i4HB—Institute for Health and Bioeconomy at Instituto Superior Técnico, Universidade de Lisboa, Av. Rovisco Pais, 1049-001 Lisbon, Portugal; 10 CERENA Center for Natural Resources and Environment, Instituto Superior Técnico (IST), Universidade de Lisboa, Av. Rovisco Pais, 1049-001 Lisboa, Portugal

**Keywords:** brewer’s spent grain, fibers, protein, fatty acids, sensory evaluation

## Abstract

Brewers’ spent grain (BSG), the major by-product of the brewery industry, has high nutritional value, making it suitable for upcycling into products such as healthy, and sustainable cookies. Nonetheless, the incorporation of BSG in cookies can impact their quality, given the increased fiber and protein content. This work explored the effect of replacing wheat flour with BSG at 50% and 75% in cookie formulations, focusing on physical, chemical, and sensory properties. The dietary fiber, lipid, and protein content of cookies improved considerably with the highest incorporation of BSG, increasing from 6.37% to 15.54%, 9.95% to 13.06%, and 9.59% to 12.29%, respectively. Conversely, moisture and water activity decreased from 11.03% to 3.37% and 0.742 to 0.506, respectively, forecasting a lower risk of microbial contamination and increased shelf-life. The incorporation of BSG in cookies resulted in decreased brightness and increased hardness, from 40 N to 97 N. Moreover, colorimetric shifts among the control cookies and the two BSG-rich formulations could be easily identified by an untrained observer. Sensory evaluation showed that cookies with 50% BSG retained acceptable sensory characteristics, suggesting potential for further development. Overall, BSG enhances the nutritional profile of cookies with no excessive detrimental impact on sensory features.

## 1. Introduction

The development of sustainable production methods to reduce waste and minimize environmental impact has become a hot topic in the food industry in recent years [1,2,3,4]. As well as this, consumers have become increasingly aware of the close connection between diet and health [5,6,7]. This has fueled the quest for foods that not only satiate hunger but also promote health. Concomitantly, the food sector has actively engaged in developing and designing foods that incorporate health-enhancing ingredients [8,9]. Moreover, significant efforts have been made to include in this trend the basic principles of circular bioeconomy, so that resources are efficiently used, waste is minimized, and natural systems are regenerated [10,11]. Circular bioeconomy in the food sector fosters the valorization of by-products generated throughout production, thus not only addressing environmental concerns but also adding nutritional value to food formulations through the incorporation of said by-products [1,4,12,13,14]. One such by-product is spent grain [15,16,17].

Large amounts of brewers’ spent grain (BSG) are generated in brewing processes [18]. This material is typically considered waste [19] and has traditionally been discarded as animal feed, organic fertilizer, and for brick production [15,18,19]. Yet, BSG is rich in fiber (mostly cellulose and hemicellulose), proteins, lipids, minerals, (poly)phenols, and vitamins [18,19,20]. These classes of compounds, thus, constitute indispensable components of the human diet, as well as molecules that are prone to bring health benefits [1,19]. These nutritional features make BSG a sound candidate to be incorporated into foods to deliver products with improved nutritional quality and appeal to the consumer [1,16,17,19,21]. Moreover, this pattern is eased even when regulatory issues are considered since the ingredients used in brewing are approved for human feeding [19]. Notwithstanding, the impact of incorporating spent grain in foods on features such as sensory and physical properties and shelf life of the final product needs to be carefully assessed [18,19]. Overall, too low an incorporation of BSG in baked goods (under approximately 5% *w*/*w*) has relatively negligible effects, whereas too high an incorporation (above approximately 20% *w*/*w*) may have a negative impact on the functional properties and sensory profile of the baked good, ultimately affecting consumer acceptability, as summarized elsewhere [18,22,23,24]. Considering these matters, it is not surprising that in recent years several works have focused on evaluating the use of BSG to deliver food products with increased nutritional value and adequate physicochemical properties. Recent examples of such food products where at least one of those features was assessed include bakery goods [25,26,27,28], bread [21,29,30,31,32,33,34,35], cereal-based beverages [35], chip-type products [36], meat products [37,38], pasta [39,40,41,42,43,44], and snacks [45]. Further examples can be found in dedicated reviews, e.g., [1,17,19]. Still, it is well-known that various industrial activities and environmental phenomena are accountable for soil, air, and water pollution, resulting in an increase in the concentration of heavy metals in the soil. This increase can lead to the absorption of metals by agricultural crops, leading to contamination of the food chain, which could have negative impacts when the incorporation of BSG in foods is considered. It is therefore essential to monitor the elemental composition at all stages of the production process, from the raw materials to the final product [46].

The present work aims to further contribute to the current knowledge on the characterization of BSG-enriched food products. Specifically, this work addresses the use of BSG in cookie production, focusing on its impact on physical, nutritional, and sensory attributes. Thus, it explores how incorporating BSG into cookies can enhance their nutritional profile, improve sustainability by utilizing a byproduct of the brewing process, and maintain or enhance sensory attributes such as taste, texture, and aroma. Moreover, the work promotes the valorization of BSG as a valuable ingredient in the food industry while offering consumers delicious and nutritious cookie options.

## 2. Materials and Methods

### 2.1. Preparation of BSG

Wet BSG was collected from the brewery Cervejaria Crafters (Sintra, Portugal) obtained after the brewing process. The source of BSG was based on the brewing of barley malt and is the remains after the extraction of the wort, and before fermentation. The BSG was refrigerated immediately after collection and then dried in an oven with forced air circulation (J.P. Selecta, Barcelona, Spain) at 50 °C until the weight stabilized to enhance the shelf life, reduce the volume, and ease material handling. The dried sample was milled in a domestic blade grinder (Moulinex, Alencon, France), sieved through 400 µm mesh, packed, sealed in polyethylene bags to prevent oxygen exposure, and stored at −20 °C away from light until analysis [47].

### 2.2. Cookies Preparation

Three cookie formulations were prepared: control cookie (CC) with wheat flour, BSG-modified cookies in which wheat flour was replaced by 50% *w*/*w* (CSG50), and 75% *w*/*w* (CSG75) BSG. The cookie recipe formulation is shown in Table 1.

Eggs, sugar, and melted butter were added to a large bowl and whisked together by hand for about 2 min, after which olive oil was added. The flour was then slowly added so that no lumps were formed. For CSG50 and CSG75 formulations, BSG and cinnamon were the added. The mixture was thoroughly mixed in a food processor (Vorwek, Wuppertal, Germany) at position 4 for 10 min until a consistent and homogeneous dough was obtained. The yogurt and green lemon zest were stirred in. Tablespoonfuls then dropped small rounds of the dough onto a tray lined with parchment paper and baked in a preheated forced air convection electric oven (eka KF933, Padova, Italy) at 150 °C for 20 min until the cookies were golden and crispy.

### 2.3. Elemental Analysis

The total metal concentrations in BSG were determined as described elsewhere [48]. Briefly, after sample digestion with nitric acid in a microwave oven, the concentrations of the metals were determined using atomic absorption spectrometry (Solaar-Thermo Elemental Thermo Fisher Scientific, Inc., Cambridge, UK) with flame mode applied for Cd, Cr, Cu, Fe, Mn, Ni, Pb, and Zn. Quantification was based on a calibration curve generated from external standards. Arsenic levels were assessed using a hydride generator, where sodium borohydride was added to convert As(III) into a volatile hydride (Solaar-Thermo Elemental-VP90 Continuous Flow vapor Accessory). This hydride was then removed from the solution by purging it with a stream of argon gas. Again, a calibration curve derived from external standards was used for accurate measurements. Mercury concentrations were measured directly in samples using atomic absorption spectrometry with thermal decomposition, employing a Direct Mercury Analyzer (DMA Milestone, Sorisole, Italy). Control samples were prepared employing an identical procedure, omitting the inclusion of a test sample. The analyses were duplicated, meeting the quality criterion for duplicates, which required a variation of less than 15%. All the reagents used were Merck Suprapure quality, and MilliQ grade water was used. Working metal solutions were prepared using 1.0 × 10^−3^ mg/L standard solutions (Merck, Darmstadt, Germany).

### 2.4. Moisture Content

Moisture content was determined in triplicate (approximately 5.0 g samples) using a PMB-202 moisture analyzer (AE Adam Equipment, Milton Keynes, UK) and the results were expressed as a sample percentage (%) [48,49].

### 2.5. Ash Content

Ash content was determined in triplicate as described elsewhere [48,49]. Briefly, 2 g samples were placed in an oven (Selecta, Barcelona, Spain) with forced air circulation at 105 °C for 2 h. Afterward, samples were placed in a muffle furnace (J.P. Selecta-Horn, Barcelona, Spain) at 550 °C for 4 h. Ash content was calculated according to Nicolai et al. [49] and the results were expressed as a sample percentage (%).

### 2.6. Dietary Fibers

Total dietary fibers (TDFs) were determined for cookies by an enzymatic gravimetric protocol based on the AOAC method 985.29 and according to Monteiro et al. [50] using the K-TDFR-200A Megazyme kit (Neogen Europe Ltd., Ayr, Scotland) as specified by the method 2 kit supplier.

The cookie samples were dried in an oven at 105 °C overnight and cooled in the desiccator. In a 400 mL precipitation beaker, about 1.0 g of dried sample was weighed in duplicate. Then, 50 mL of phosphate buffer (pH 6.0) was added. Subsequently, the samples were gelatinized by incubation with α-amylase thermostable and then enzymatically digested with protease and amyloglucosidase to remove protein and starch which was followed by two incubations in a thermostatic bath with stirring (50 rpm) (J P Selecta, Unitronic 320, Barcelona, Spain). Then, the total dietary fiber was precipitated with 280 mL 96% ethanol, followed by filtration of the residue using a vacuum pump (Gast, model D0A-P104-BN, Benton Harbor, MI, USA). Finally, the residue was washed with 78% ethanol, 95% ethanol, and acetone. The dried residue was weighed, and a sample duplicate was analyzed for protein determination (Kjeldahl method), whereas another was incinerated at 525 °C for 5 h for ash content determination. The total dietary fiber corresponds to the mass of the residue in percentage after digestion minus the value for protein and ash, also rectified by subtracting the value of the blank test [50].

The determinations of soluble dietary fiber (SDF) and insoluble dietary fiber (IDF) in BSG were performed with the same K-TDFR-200A Megazyme kit by AOAC method 991.42, with the following modification to the kit supplier method 1: phosphate buffer (50 mL, 0.08 M, pH 6.0) was used for 1 g of BSG dry sample. The total dietary fiber (TDF) was calculated as the sum of SDF and IDF. TDF, SDF, and IDF results were calculated in sample mass percentages.

### 2.7. Total Lipidic Content

Roughly 5.0 g of BSG (processed as described in Section 2.1) was weighed onto a Soxhlet extraction thimble. The sample was then transferred to a Soxhlet extractor with a distillation flask containing 200 mL ether petroleum and extracted continuously for eight hours. The solvent was eliminated by evaporation at room temperature, and the lipid content was quantified by weighing.

### 2.8. Protein Content

The determination of protein content in 0.5 g samples was carried out by the Kjeldahl method [51] using automated distillation and titration (Foss, 2300 Kjeltec analyzer unit, Hillerød, Denmark). Samples were previously digested in sulfuric acid with a catalyst (potassium sulfate and copper sulfate) with a temperature increasing to 430 °C during 3 h in a digestor (Foss, Tecator 2020 Digestor, Hillerød, Denmark). Then, automated distillation was performed with sodium hydroxide (50%) and distilled ammonia, using bromocresol green and methyl red as indicators in reaction with boric acid (2%), followed by hydrochloric acid (0.10 N) titration. The crude protein content was calculated using a conversion factor of 6.25 to convert the determined nitrogen percentage to the crude protein percentage [50].

Duplicate measurements were carried out and the results were expressed as a sample percentage (%).

### 2.9. Energy Content

To estimate the energy content, the available carbohydrates in % were calculated by difference, subtracting the determined percentages of moisture, ash, dietary fiber, fat, and protein, from 100% as in FAO 2003 [52].

The energy evaluation, expressed in kJ/100 g, was calculated using conversion factors based on the general Atwater factors for food energy content as detailed in EU Regulation N. 1169, 2011 [53] and Monteiro and co-workers 2022 [50,52]. The factors used were 17 kJ/g for carbohydrates and protein, 8 kJ/g for fiber, and 37 kJ/g for fat [50,52].

### 2.10. Total Phenolic Content

The total phenolic content was determined spectrophotometrically according to the Folin−Ciocalteu method [54] and expressed as mg of gallic acid equivalent (GAE) per gram of dry matter (BSG or cookie) [49]. Briefly, 1 g of grounded cookie was extracted with 10 mL of 60% aqueous ethanol using an Ultra-Turrax homogenizer for 1 min (Ultra-Turrax T25, IKA Labortechnik, Staufen, Germany). The suspension was then incubated in an ultrasonic bath (Bransonic, Branson 5200, Branson, MO, USA) for 40 min at 40 kHz and then centrifuged (10 min, 7000 rpm, 4 °C, Sigma, model 2K15 with rotor 12139-H, Osterode am Harz, Germany) according to Pereira et al. 2023 [55] with some modifications.

Samples of the extract (150 μL) were introduced into test tubes, and 2.4 mL of ultrapure water, and 150 μL of Folin–Ciocalteu’s reagent (0.25 M) were added followed by vortex shaking (Heidolph, Reax top, Staufen, Germany) and 3 min incubation at room temperature in the dark. Afterwards, 300 μL of sodium carbonate (0.5 M) was added, and the tubes were mixed and allowed to stand in the dark for 2 h [56]. Absorption at 725 nm was measured (Jasco V-530 UV/VIS Spectrophotometer, Tokyo, Japan), and quantification was performed using a calibration curve previously established with gallic acid as standard (0.01 to 0.25 mg/mL).

### 2.11. Physical Properties

The water activity (A_w_) at 20 °C was determined in triplicate using a LabMaster-aw neo (Novasina^®^, Lachen, Germany) [57].

Colorimetric properties were measured on the surface of the cookies using a previously calibrated Konica Minolta CR-400 portable colorimeter (Osaka, Japan) as described elsewhere [58]. The measurements were performed in triplicate for each formulation at 25 °C, under consistent artificial lighting, at various time points. The results were presented using the CIELAB system that establishes a 3-dimensional color space based on three parameters, L*, a*, and b*; here, L* is a vertical axis that defines brightness and a* and b* are perpendicular horizontal axes that define red-to-green and blue-to-yellow, respectively [58,59]. In addition, chroma (C*) and hue angle (h°) were calculated from a* and b* according to Equations (1) and (2)

(1) $$C^{*}={\left({a^{*}}^{2}+{b^{*}}^{2}\right)}^{0.5}$$

(2) $$h{}^{\circ}={tan}^{-1}\left(\frac{b^{*}}{a^{*}}\right)$$

respectively [58,59]. The total difference in colorimetric properties (ΔE) was calculated according to Equation (3) [58].

(3) $$∆E={\left[{\left(∆L^{*}\right)}^{2}+{\left(∆a^{*}\right)}^{2}+{\left(∆b^{*}\right)}^{2}\right]}^{0.5}$$

The hardness of the cookies was assessed by conducting a puncture test using a texture analyzer TA.XT plusC (Stable Micro Systems, Godalming, UK) fitted out with a cylindrical probe (SMSP/2SP) with a 2 mm diameter. Replicate measurements, 20 times per formulation (1 in each cookie), were carried out in a room with a controlled temperature (20 °C, three days after preparation), after baking and cooling. Measurements were performed with a test speed of 1 mm/s and 4 mm penetration depth. Hardness is expressed as the maximum force (N) needed when penetrating a probe into the cookie to the pre-defined depth.

### 2.12. Sensory Analysis

The sensory analysis was conducted in the sensory analysis laboratory of INIAV (UTI, Oeiras, Portugal), using individual tasting booths designed in compliance with ISO 8589:2007 standards [58,60]. The panel consisted of 24 semi-trained participants (83.3% female, 16.7% male; age range: 15–63 years), representing a diverse group with varying levels of familiarity with cookies, capturing a broad spectrum of potential consumer preferences.

To ensure consistency and reliability, the evaluation was conducted in two independent sessions with the same panel under identical conditions. Formulations CC (control), CSG50 (50% BSG substitution), and CSG75 (75% BSG substitution) were simultaneously presented to panelists in both sessions. The samples were randomized in order and identified by three-digit codes. Panelists assessed the cookies based on appearance, color, flavor, texture, and overall appreciation, using a five-point hedonic scale (1 = “very unpleasant” to 5 = “very pleasant”). Additionally, participants indicated their purchase intention for each formulation. To minimize taste fatigue and ensure reliable evaluations, panelists rinsed their mouths with water between samples.

This study was conducted in full compliance with ethical and data protection standards. All procedures adhered to the EU General Data Protection Regulation (EU 2016/679) [61] and the INIAV Code of Ethics and Conduct, approved by its Board of Directors on 7 January 2019. Participants were fully informed about the study’s objectives and voluntarily provided their informed consent prior to participation. While the sensory analysis posed minimal ethical risks, rigorous measures were implemented to protect participant confidentiality and ensure the security of collected data.

### 2.13. Statistical Analysis

The data are presented as means ± standard deviation (SD) for continuous variables and as absolute and relative frequencies (%) for categorical variables. Independent sample Student’s t-test was used to compare the means of variables. One-way ANOVA was used for comparing means across more than two groups, followed by Tukey’s Honest Significant Difference (HSD) test. Statistical analysis was carried out using Statistica 7.0 (StatSoft. Inc., Tulsa, OK, USA). Results were deemed statistically significant at *p* < 0.05.

## 3. Results and Discussion

### 3.1. Characterization of BSG and BSG-Rich Cookies

#### 3.1.1. Elemental Analysis of BSG

The presence of heavy metals in agricultural by-products like BSG raises significant health concerns, necessitating a comprehensive evaluation before their inclusion in human diets. Toxic heavy metals, such as arsenic, cadmium, lead, and mercury, are known for their harmful effects and their ability to bioaccumulate in the food chain, leading to serious health risks for humans and animals [62,63,64]. Although arsenic is a metalloid, its environmental behavior and toxicity—especially in its inorganic and carcinogenic forms—justify its classification alongside heavy metals [62,65,66]. Research has shown that heavy metal contamination in agricultural products can occur through various sources, such as soil pollution from industrial activities, mining, and agricultural runoff [67,68]. Contaminated soils, for instance, can result in heavy metal accumulation in crops, posing a direct risk to the human food chain [69].

The bioaccumulation of these metals in food, including grains, can contribute to long-term health issues like neurological damage, reproductive toxicity, and an increased risk of cancer [70,71]. Therefore, it is essential to assess the specific concentrations of heavy metals in BSG from suppliers (Table 2) to ensure their safety for human consumption. Monitoring heavy metal levels in agricultural by-products is vital to comply with health and safety standards, as highlighted in recent studies [72]. Additionally, the application of advanced detection methods and risk assessment strategies can help identify these contaminants and evaluate their potential health impacts [73,74].

As expected, BSG also contained essential minerals, such as iron, manganese, and zinc [75,76]. These minerals contribute to bone mineralization and are co-factors for antioxidant enzymes and vitamins. Moreover, iron aids in oxygen transportation and helps prevent anemia, and manganese supports metabolism, hematopoiesis, endocrine regulation, immune function, and cartilage formation [77].

In summary, incorporating BSG into the human diet requires careful examination of elemental content. The potential health risks from exposure to these contaminants necessitate strict testing and monitoring to protect public health. Various studies underscore the importance of addressing heavy metal contamination in food sources to reduce health risks and ensure food safety [62,63,64,65]. Still, under careful monitoring, including BSG in food sources can help address iron, manganese, and zinc deficiencies and thus promote overall health.

#### 3.1.2. Nutritional Composition

The proximate composition of both BSG and cookies is depicted in Table 3.

The moisture content of the cookie formulations is noticeably under 15%, therefore providing an environment that is considered to prevent the risk of microbial food spoilage [78,79]. This trend is further enhanced as the BSG titer increases. As in the present case, a decrease in the moisture of cookies upon baking due to BSG incorporation was previously reported [27,80,81]. In any case, moisture was under 40%, which may suggest that a gluten network was not formed [27].

The ash content of BSG used in this work falls within the typical range of 2.4 to 4.6% reported in the literature [18,75] and is significantly lower than that observed by Ajanaku and co-workers, approximately 17% [82]. The latter is unusual and could be due to the high mineral content of the grains [83], possibly due to contamination with soil or dust, among other environmental conditions [84]. Since the ash content of BSG exceeded that of the control cookies, increasing BSG incorporation in cookies led to a concomitant increase in ash content, yet still by far within the range of ash content of food [85]. The higher ash content of the supplemented cookies suggests a higher mineral content when compared to the control cookies [86], which was expected since the typical ash content in wheat flour ranges from 0.5% to 1% [87].

The total dietary fiber content of BSG, approximately 41%, falls within the range of 30% to 50% reported in the literature [18] and exceeds the total dietary fiber content of wheat flour, which ranges from 9 to about 20% [88]. The dietary fiber in BSG is mostly insoluble, comprising 37.3% insoluble fiber and only 4.04% soluble fiber. This is likely to improve gut immunity and intestinal integrity, enhance mucosal growth, and promote probiotic adhesion, overall furthering human health [89].

The observed lipid content of BSG, approximately 4.45%, falls within the range of 3% to 13% reported in the literature [18], and exceeds the typical lipid content in wheat flour, which is reported in the literature to range from 0.8% to 2.9% [87,90]. The gradual incorporation of BSG in cookies led to a concomitant increase in the lipid content when compared to the control cookies, up to approximately 13.1%, which is close to the typical fat content of cookies, 14.3% [91].

The protein content of BSG used in the present work follows that reported in the literature [92]. This was expected since it is well established that protein, as well as fiber, has high titers in BSG since most of the starch is depleted during mashing [75]. Protein content in BSG also exceeded that typically found in wheat flour, which is about 8 to 11% [58]. Therefore, the increased protein content observed as BGS is gradually incorporated into the cookies, compared to the control, was expected and contributed to an increase in the nutritional value of the cookies [93]. The same trend was reported by Petrovic and co-workers [25], but higher protein content in BSG-rich cookies was observed in the present work. Still, this last outcome may have also been influenced by higher protein content in the control cookies compared to those used in said work. Nevertheless, beyond protein content, the type of protein plays a crucial role in determining an ingredient’s suitability for bakery applications. The elastic properties of glutenins, which form the gluten network in wheat, are absent in other cereals and BSG, impacting the functional performance of these alternatives in baking.

#### 3.1.3. Phenolics Concentration

Phenolic compounds have been related to positive impacts on human health. This has been primarily related to the antioxidant activity of phenolic compounds and tentatively to the interaction of phenolic compounds with the intestinal microbiota and the resulting impact on the so-called gut-brain axis [94]. BSG is a good source of phenolic compounds; hence, the incorporation of BSG in baked goods is likely to deliver a product with added BSG is expected to improve the titer of polyphenolic compounds, which was observed (Table 4). The total phenolic content of BSG was 1.27 ± 0.02 mg GAE/g of dry matter, showing a significant difference compared to the control cookies but not when compared to the TPC of the cookies incorporating BSG.

The total phenolic content of BSG-enriched cookies consistently bested that of the control cookies, a pattern also reported by Baiano and co-workers when addressing the incorporation of BSG in bread [21].

### 3.2. Physical Properties of BSG-Rich Cookies

#### 3.2.1. Water Activity

Water activity, alongside moisture content, are key parameters to assess the quality of food products, as both affect consistency, weight, contamination risk, and shelf-life, among other factors [95].

In this study, water activity in cookies consistently decreased as BSG incorporation increased (Table 5), following the trend previously observed in reduced moisture levels.

A similar decrease in water activity with increased BSG incorporation in chips was reported by Garrett and co-workers [36]. Overall, the decrease in both moisture content and water activity due to BSG incorporation plays a key role in minimizing microbial contamination risk and extending the product’s shelf-life [80].

#### 3.2.2. Color and Hardness

The color of the food is the initial feature of quality assessed by consumers [96]. The incorporation of BSG in cookies at different titers led to noticeable color differences as quantitatively assessed (Table 6).

The incorporation of BSG into cookie formulations led to a noticeable decrease in brightness (L* values), with a reduction of approximately 30%, resulting in darker cookies compared to the control. The darker, brownish hue in BSG-enriched cookies is attributed to the Maillard reaction involving the BSG proteins and reducing sugars [25,97].

In terms of chromaticity, the a* value (red-green axis) roughly doubled with BSG incorporation, although this increase showed no detectable differences between CSG50 and CSG75. These shifts in colorimetric coordinates are highlighted by findings from previous reports where the impact of BSG incorporation in baked goods was addressed [25]. The b* value (yellow-blue axis) also consistently decreased, indicating a shift towards blue as BSG levels increased. This trend was also reported by Petrovic and co-workers, who observed a slight decrease in the b* value at 50% BSG incorporation, though they found no significant changes with lower BSG levels and did not test higher levels [25]. Garret and co-workers also reported a consistent decrease in the b* value in chips with BSG incorporation from 8% to 40% [36]. On the other hand, Vriesekoop and co-authors reported an increase in the b* value in sourdough bread with BSG incorporation, potentially due to their use of solid-state fermented BSG [98]. Additionally, both h° (hue angle) and C* (chroma) values decreased with BSG incorporation, indicating a shift in overall color intensity. Calculated ΔE values further revealed discernible color differences (ΔE much higher than 5) among each BSG-enhanced cookie formulation and the control ($∆E_{with\;CC}^{*}$), as well as between formulations, which would be noticeable even to untrained observers [99]. However, the color differences between the BSG-enriched formulations (3 < ΔE < 5) are detectable only by experienced tasters.

#### 3.2.3. Hardness

**Figure 1:**
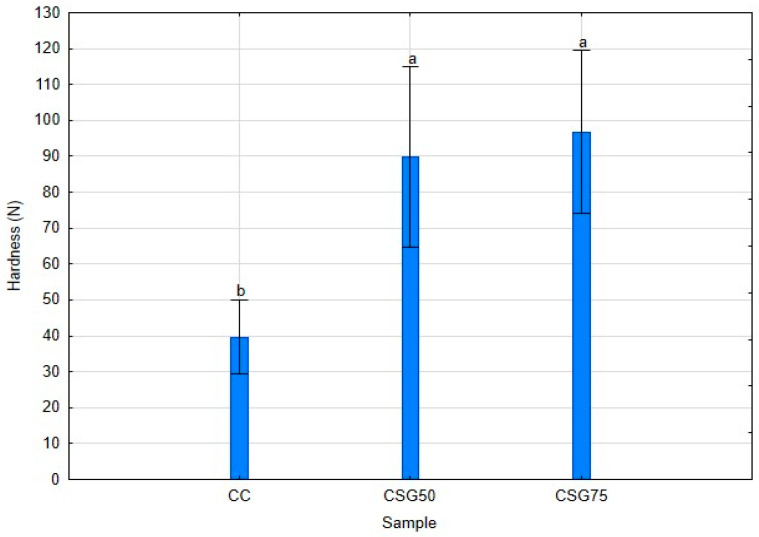
Effect of BSG incorporation on cookie hardness. In the Figure, CC stands for control cookies baked with wheat flour, CSG50 and CSG75 refer to cookies in which wheat flour was replaced with 50% (*w*/*w*) and 75% (*w*/*w*) BSG, respectively. Mean values and standard deviation (error bars) of *n* independent assays; the same superscript letter means no significative differences (ANOVA, Tukey HSD test, *p* < 0.05, *n* = 20).

As wheat flour, the major component in traditional cookies, was gradually replaced with BSG, cookie hardness increased significantly (*p* < 0.05) (Figure 1).

The increase in cookie hardness with higher levels of BSG incorporation can be attributed to the distinct composition differences between BSG and wheat flour. Wheat flour is high in starch (around 70–75%) and has a relatively low protein content (8–11%) [100]. This high starch content contributes to a softer, more tender crumb structure in baked goods, as starches gelatinize, and provide a soft texture upon baking. Additionally, the gluten proteins in wheat flour (such as glutenins and gliadins) provide elasticity and structure, contributing to the typical texture of cookies.

In contrast, BSG is rich in protein (approximately 20%) and fiber (approximately 70%) [101], and these components significantly impact texture. The high fiber content of BSG introduces a denser matrix, as fibers do not soften like starches and instead create a more rigid structure in the baked product. This increased fiber level also reduces moisture retention (Table 3) which can further contribute to a firmer texture. The proteins in BSG, although higher in content, lack gluten-forming properties, which are critical for creating the elasticity that softens the texture. As a result, the protein in BSG does not contribute to the flexible network gluten provides in wheat flour but instead adds more rigidity [15,27,98].

Thus, as BSG levels increase in the cookie formulation, this results in a more compact and less elastic structure, leading to the observed increase in hardness. This outcome aligns with previous findings where BSG incorporation in baked goods also resulted in increased hardness due to similar compositional effects. The significant increase in cookie hardness (*p* < 0.05) aligns with the expected texture profile when wheat flour is replaced with a high-protein, high-fiber ingredient like BSG [15,58]. However, there are no significant differences in hardness between the 50% and 75% (*w*/*w*) BSG formulations.

### 3.3. Sensory Evaluation of BSG-Rich Cookies

**Figure 2:**
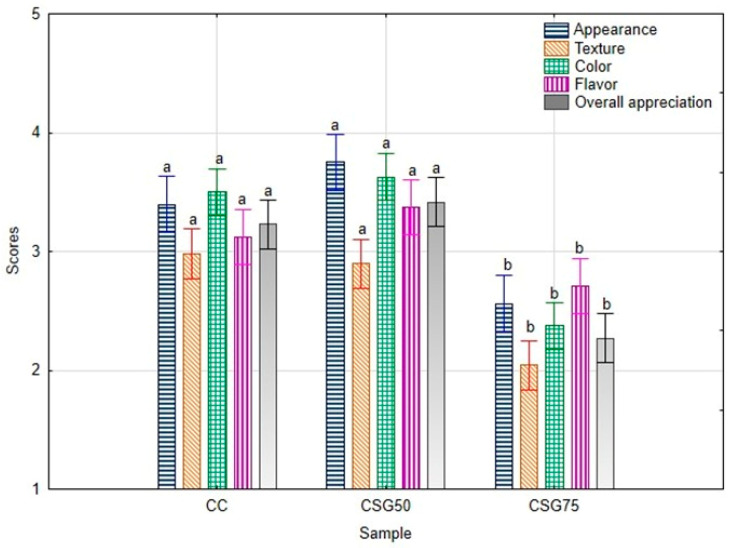
Sensory evaluation of cookies with BSG as a wheat flour substitute using the 5-point hedonic scale. In the Figure, CC stands for control cookies baked with wheat flour, CSG50 and CSG75 refer to cookies in which wheat flour was replaced with 50% (*w*/*w*) and 75% (*w*/*w*) BSG, respectively. Data are presented as mean of *n* = 24 responses and *n* = 2 sessions replicates. Different letters indicate significant differences, according to the ANOVA followed by Tukey’s test (*p* < 0.05).

The sensory evaluation was conducted to assess the appearance, color, flavor, texture, and overall appreciation of cookies formulated with brewers’ spent grain (BSG) as a partial substitute for wheat flour (Figure 2).

The results demonstrate that cookies made with 100% wheat flour (control cookies, CC) consistently received good scores (higher than 3) across all evaluated attributes, which is consistent with previous studies that indicate a preference for traditional wheat-based products [102]. Among the BSG-substituted formulations, the cookies with 50% BSG substitution (CSG50) generally received higher sensory ratings compared to those with 75% BSG substitution (CSG75). A noticeable decrease in appearance and color scores was observed as the level of BSG substitution increased. This decline is likely due to the darker color imparted by the BSG, which has been reported to reduce the visual appeal of baked products [102]. Despite this, the CSG50 formulation retained relatively favorable scores for these attributes, indicating that the panel found the visual appeal acceptable even with the 50% BSG inclusion. In contrast, the CSG75 formulation received lower appearance and color scores, likely due to the more pronounced color change from the increased BSG content. Texture scores followed a similar trend, with CSG75 receiving the lowest ratings. Higher levels of BSG substitution can alter the structural integrity of the dough due to its higher fiber content, resulting in a coarser and drier texture. In terms of flavor and overall appreciation, the control cookies (CCs) scored the highest, with the CSG50 formulation receiving moderate ratings. However, the CSG75 cookies were the least favored, reflecting a decrease in flavor and overall acceptance as the BSG substitution level increased. This result echoes previous research by Heredia-Sandoval and co-authors [103], where a higher BSG content led to less favorable flavor profiles, likely due to the bitter or astringent aftertaste associated with increased fiber content in BSG.

The purchase intention test indicated that the panel would possibly purchase CC (standard cookie) and CSG50, as the average purchase intention scores were close to 66% and 68%, respectively. This preference may be attributed to the balance between incorporating the nutritional benefits of BSG and maintaining a favorable sensory profile [104]. As for the CSG75 sample, only 20% of the tasters showed interest in purchasing.

Overall, the results suggest that while BSG can be successfully incorporated into cookie formulations, the substitution levels should be carefully considered. The sensory evaluation supports the use of up to 50% BSG substitution to maintain consumer acceptance, particularly regarding appearance, texture, flavor, and overall appreciation. Higher substitution levels, such as 75%, may negatively impact sensory attributes, especially texture and flavor, and could limit the acceptability of the product among consumers. The comparison between instrumental color data and sensory panel results is evident in the observed data. As instrumental measurements showed a significant decrease in brightness (L* values) and shifts in chromaticity (a* and b* values), these changes were reflected in the sensory panel’s lower ratings for appearance, color, and overall acceptability. The darker appearance likely made the cookies less visually appealing to the panelists. The increase in a* values and decrease in b* values indicated a more intense and less yellow hue, contributing to the less appealing appearance noted by the sensory panel. Understanding this correlation makes it clear that while BSG incorporation offers nutritional benefits, it also introduces noticeable color changes that can affect consumer acceptance. Incorporating BSG results in increased hardness and a less favorable texture, particularly for the BSG75 sample, which received a score of 5. This underscores the influence of BSG on the cookies’ physical and sensory attributes. However, despite the increased hardness measured by the texturometer in the BSG50 sample, the texture score did not show a significant difference compared to the control sample. In general, to successfully launch a product in the market, it is recommended that the sensory score for the product should be at least 3 (“like”) on the hedonic scale [105]. Based on this criterion, the CSG50 formulation, which received higher sensory scores, could be considered for further development and potential market introduction.

Furthermore, the buying preferences of the panelists were also assessed. The results indicated that 88% of the panelists expressed a preference for the control sample, followed by 53% showing interest in purchasing the CSG50 formulation. In contrast, only 17% of the panelists indicated that they would consider buying the CSG75 formulation.

If a health claim were made highlighting the nutritional benefits of BSG in the CSG50 formulation, such as its high fiber content and potential positive effects on gut health, it could lead to a significant increase in consumer preference. Health-conscious consumers are often willing to overlook minor differences in sensory attributes if the product offers enhanced health benefits. By marketing CSG50 as a healthier alternative, the formulation could potentially attract more buyers, increasing its appeal and preference beyond that of the control sample, particularly among those prioritizing health in their food choices.

## 4. Conclusions

The results obtained in this work confirm that BSG can effectively replace wheat flour at significant levels, namely 50% and 75%, to deliver an edible good with enhanced nutritive value and acceptable sensory properties. The partial replacement of wheat flour with BSG in cookies ultimately resulted in a 2.4-fold increase in dietary fibers, and a 1.3-fold increase in lipids and proteins, enhancing nutritional benefits such as improved digestive health, energy content, texture, flavor, and satiety overall, contributing to a more balanced diet. Hardness roughly increased 2-fold, improving structural integrity and suggesting improved endurance to packaging, transportation, and storing, increasing durability. Increased hardness also minimizes moisture absorption, which was shown to be reduced up to 3.7-fold. Water activity also decreased up to 1.3-fold, further reinforcing the perspective of improved shelf-life and lower risk of microbial contamination. Additionally, phenolic content increased 1.5-fold, which is likely to have a positive impact on the gut–brain axis. The present work highlighted several limitations associated with the incorporation of BSG into cookies. A negative impact on the sensory qualities of the product was observed, including a darker color and increased hardness, both of which can reduce consumer acceptability. These effects were particularly pronounced at a 75% incorporation level. Although the addition of BSG enhances the nutritional profile of cookies, it compromises critical functional properties, such as texture and water activity. Moreover, when using this type of flour, careful monitoring of heavy metal content is essential to ensure compliance with regulatory standards. Cookies, where 50% wheat flour was replaced with BSG (CSG50), received the best balance of sensory scores and consumer interest, making them a strong candidate for market development. Given their health benefits, such as high fiber content, BSG-rich cookies can thus attract health-conscious consumers, despite minor sensory compromises. 

## Figures and Tables

**Table 1:** Cookie recipe formulation. The ingredients were weighed based on a 150 g batch. In the Table, CC stands for control cookies baked with wheat flour, CSG50 and CSG75 refer to cookies in which wheat flour was replaced with 50% (*w*/*w*) and 75% (*w*/*w*) BSG, respectively.

Ingredients	CC	CSG50	CSG75
	(g)		
Wheat flour (T65 without yeast, Nacional, Portugal)	150.0	75.0	37.5
Table sugar (Pingo Doce, Portugal)	50.0	50.0	50.0
BSG (Sintra, Portugal)	0	75.0	112.5
Banana yogurt (Porsi Intermarché, Portugal)	125	125	125
Fresh eggs (free-range chickens, Matinado, size M/L, Pingo Doce, Portugal)	70	70	70
Extra virgin olive oil (Continente, Portugal)	4.0	4.0	4.0
Butter (Mimosa, Portugal)	25.0	25.0	25.0
Cinnamon powder (Margão, Portugal)	2.0	2.0	2.0
Green lemon zest (Continente, Portugal)	3.0	3.0	3.0

**Table 2:** Elemental composition of BSG from Cervejaria Crafters.

Metals	Concentration (mg/kg)
As	<0.05
Cd	<0.50
Cr	<5.0
Cu	9.1 ± 1.4
Fe	115 ± 17
Hg	<0.0083
Mn	56.5 ± 5.1
Ni	<7.5
Pb	<10
Zn	64.9 ± 9.6

**Table 3:** Nutritional composition of BSG and cookies formulations. In the Table, CC stands for control cookies baked with wheat flour, CSG50 and CSG75 refer to cookies in which wheat flour was replaced with 50% (*w*/*w*) and 75% (*w*/*w*) BSG, respectively.

Component	BSG	CC	CSG50	CSG75
Moisture (%)	6.00 ± 0.20 ^b^	11.03 ± 1.03 ^a^	7.00 ± 0.36 ^b^	3.37 ± 0.63 ^c^
Ash (%)	2.57 ± 0.23 ^a^	0.75 ± 0.00 ^c^	1.15 ± 0.05 ^b^	1.33 ± 0.03 ^b^
Total dietary fiber (%)	41.29± 0.12 ^a^	6.37 ± 0.09 ^d^	8.76 ± 0.54 ^c^	15.54 ± 0.52 ^b^
Lipids (%)	4.45 ± 0.03 ^d^	9.95 ± 0.04 ^c^	12.22 ± 0.01 ^b^	13.06 ± 0.01 ^a^
Protein (%)	15.71 ± 0.28 ^a^	9.59 ± 0.01 ^d^	11.53 ± 0.03 ^c^	12.29 ± 0.02 ^b^
Carbohydrates (%)	30.0 ± 0.8 ^d^	62.3 ± 1.1 ^a^	59.3 ± 0.9 ^b^	54.4 ± 1.0 ^c^
Energy (kJ/100 g)	1272 ± 7 ^c^	1641 ± 17 ^b^	1727 ± 10 ^a^	1742 ± 14 ^a^

Mean values ± standard deviation of *n* independent assays; the same superscript letter in a line means no significative differences (ANOVA, Tukey HSD test, *p* < 0.05, *n* = 3).

**Table 4:** Impact of BSG incorporation in the cookie’s phenolic content. In the table, CC stands for control cookies baked with wheat flour, CSG50 and CSG75 refer to cookies in which wheat flour was replaced with 50% (*w*/*w*) and 75% (*w*/*w*) BSG, respectively.

Cookies	Total Phenolic Content (mg_GAE_/g_dry matter_)
CC	0.99 ± 0.03 ^b^
CSG50	1.47 ± 0.02 ^a^
CSG75	1.41 ± 0.15 ^a^

Mean values ± standard deviation of *n* independent assays; the same superscript letter means no significative differences (ANOVA, Tukey HSD test, *p* < 0.05, *n* = 3).

**Table 5:** Impact of BSG incorporation in the water activity of cookies. In the Table, CC stands for control cookies baked with wheat flour, CSG50 and CSG75 refer to cookies in which wheat flour was replaced with 50% (*w*/*w*) and 75% (*w*/*w*) BSG, respectively.

Cookies	Water Activity
CC	0.742 ± 0.010 ^a^
CSG50	0.642 ± 0.005 ^b^
CSG75	0.506 ± 0.020 ^c^

Mean values ± standard deviation of *n* independent assays; different superscript letter means significative differences (ANOVA, Tukey HSD test, *p* < 0.05, *n* = 3).

**Table 6:** CIELAB L*, a*, b*, C*, h° and ΔE color values for control and BSG-rich cookies. In the Table CC stands for control cookies baked with wheat flour, CSG50 and CSG75 refer to cookies in which wheat flour was replaced with 50% (*w*/*w*) and 75% (*w*/*w*) BSG, respectively.

Cookies	L*	a*	b*	h°	C*	${∆E}_{with\;CC}$	${∆E}_{with\;CSG50}$
CC	73.9 ± 1.9 ^a^	3.6 ± 1.8 ^c^	27.0 ± 2.1 ^a^	82.5 ± 3.1 ^a^	27.3 ± 2.3 ^a^	-	_
CSG50	54.1 ± 2.9 ^b^	7.5 ± 1.3 ^a^	21.9 ± 1.4 ^b^	71.3 ±2.3 ^b^	23.2 ± 1.7 ^b^	20.79	_
CSG75	53.0 ± 3.4 ^b^	6.3 ± 1.2 ^b^	17.1 ± 1.7 ^c^	69.7 ± 3.6 ^c^	18.3 ± 1.7 ^c^	23.07	4.70

Mean values ± standard deviation of *n* independent assays; the same superscript letter means no significative differences (ANOVA, Tukey HSD test, *p* < 0.05, *n* = 20).

## Data Availability

The original contributions presented in the study are included in the article, further inquiries can be directed to the corresponding author.
